# Case report: Atypical POEMS syndrome without polyneuropathy, complicated by borderline TAFRO syndrome

**DOI:** 10.3389/fmed.2024.1445971

**Published:** 2024-12-10

**Authors:** Shuai Tan, Mingyue Shang, Yasufumi Masaki, Jing Ni, Yuwei Da, Jing Sun, Yaofang Cao, Li Su, Wanling Sun

**Affiliations:** ^1^Department of Hematology, Xuanwu Hospital, Capital Medical University, Beijing, China; ^2^Hematology and Immunology, Kanazawa Medicai University, Uchinada, Japan; ^3^Department of Neurology, Xuanwu Hospital, Capital Medical University, Beijing, China

**Keywords:** POEMS syndrome, Castleman disease, TAFRO syndrome, peripheral neuropathy, ASCT

## Abstract

POEMS syndrome is a complex and rare hematological disease involving multiple physiological systems. According to the currently accepted diagnostic criteria for POEMS syndrome, polyneuropathy is one of the primary mandatory criteria. A patient presented with M protein, Castleman disease (CD), elevated vascular endothelial growth factor (VEGF), extravascular volume overload, and endocrinopathy. However, edema of the extremities hindered the diagnosis of polyneuropathy by electromyography (EMG). Eventually, we diagnosed the patient with atypical POEMS syndrome without polyneuropathy. The case also exhibited features consistent with TAFRO syndrome, such as anasarca, thrombocytopenia, and renal insufficiency. This underscores the need to emphasize that TAFRO syndrome is principally a systemic inflammatory disorder. Timely diagnosis and treatment with dexamethasone, followed by several sessions of lenalidomide and dexamethasone (Rd) regimen chemotherapy, resulted in complete remission (CR), and was followed by autologous stem cell transplantation (ASCT). This case offers valuable insights into the diagnosis and treatment of POEMS syndrome, which may prompt a reconsideration of the diagnostic criteria for this syndrome.

## Highlights

- This case provides valuable information for identifying POEMS syndrome, especially when EMG results of peripheral neuropathy may suggest severe edema. It also highlights a specific rare disease type—atypical POEMS syndrome without polyneuropathy.
- The patient, critically ill, presented with multiple rare diseases simultaneously, including POEMS syndrome, TAFRO syndrome, and Castleman disease, offering a valuable opportunity to improve disease identification.
- Rapid diagnosis and treatment (using dexamethasone) was crucial in saving the patient's life. Continued improvement in quality of life was observed with ASCT after several courses of chemotherapy using the Rd regimen, and the prognosis remains favorable to date.

## 1 Introduction

POEMS syndrome is a rare, multi-system blood disorder characterized by demyelinating peripheral neuropathy and monoclonal plasma cell proliferation (1). It has a prevalence of approximately 0.3/100000 people (2). To diagnose POEMS syndrome, the essential criteria include polyneuropathy and a monoclonal plasma cell disorder. Major criteria are sclerotic bone lesions, elevated levels of vascular endothelial growth factor (VEGF), and Castleman disease. The minor criteria include extravascular volume overload, organomegaly, endocrinopathy, skin changes, papilledema, and the presence of either polycythemia or thrombocytosis (3). POEMS syndrome is a complex and seldom-seen blood disease that can affect several bodily systems.

TAFRO syndrome, a hyperinflammation syndrome, presents with severe anasarca, thrombocytopenia, and renal insufficiency, and may or may not include Castleman disease-like lymphadenopathy upon histological examination. Primarily documented through case reports, TAFRO syndrome is an inflammatory condition. In Japan, the estimated annual incidence ranges from 110 to 502 cases, which translates to approximately 0.9 to 4.9 cases per million people (4, 5). The diagnosis of TAFRO syndrome requires three major criteria—anasarca, thrombocytopenia, and systemic inflammation—along with at least two of the four minor criteria, such as slight organomegaly and features resembling Castleman disease in lymph node biopsies (6).

While POEMS syndrome and TAFRO syndrome share features such as anasarca, organomegaly, elevated VEGF levels, and Castleman disease, their distinct pathologies are not well understood, making differentiation challenging. In certain cases, these conditions can be confused. This case presents an atypical instance of POEMS syndrome without polyneuropathy, which was also considered for a TAFRO syndrome diagnosis.

## 2 Case report

A 39-year-old male was admitted to our hospital after experiencing slurred speech and shortness of breath for the past 8 months. Two weeks prior to admission, the patient developed generalized edema, accompanied by significant pleural, abdominal, and pericardial effusion. Two days after admission, symptoms of heart failure emerged, preventing the patient from lying down comfortably, indicating a critical condition.

The patient’s medical history included hypertension and subclinical hypothyroidism, most likely related to a sudden cerebral infarction occurring 8 months earlier. Physical examination upon admission showed a temperature of 37°C, blood pressure at 144/86 mmHg, heart rate of 78 beats per minute, and respiratory rate of 22 respiratory cycles per minute. Clinical findings included gynecomastia, hyperpigmentation, and hirsutism. Palpation revealed multiple enlarged and movable lymph nodes in the cervical, axillary, and inguinal regions. The patient reported no typical clinical symptoms of peripheral neuropathy, such as numbness of the hands and feet, and glove or sock-like abnormal sensations at the ends of the extremities. This patient did not have any of the above symptoms, just a mild decrease in deep and superficial pain sensation and temperature sensation, which could be contributed to severe edema and a history of past cerebral infarction. Physical examination of the nervous system showed a mild decrease in deep and superficial pain sensation, and decreased temperature sensation; there were no significant muscle atrophy, no decreased bilateral tendon reflexes, and abdominal reflexes were normal. The patient’s bilateral Babinski’s sign was suspiciously positive. Oppenheim’s sign, Gordon’s sign, and Chaddock’s sign were not elicited. Meningeal irritation signs were negative. The patient’s muscular strength was level V− in both upper extremities and level IV+ in both lower extremities.

Laboratory tests (Table 1) revealed pancytopenia (Hb 82 g/L, WBC 2610 cells/μl, PLT 67,000 cells/μl), elevated CRP level at 57 mg/L, creatinine level at 150 μmol/L, uric acid at 452 μmol/L, and a B-type natriuretic peptide level of 3,618 pg/ml. The serum interleukin-6 (IL-6) level had increased to 23.23 pg/ml, and the serum vascular endothelial growth factor (VEGF) level was 330.69 pg/ml, exceeding the normal range of 0–142 pg/ml. The patient’s thyroid-stimulating hormone (TSH) level was elevated at 5.37 μIU/ml, with reduced free T3 (FT3) and free T4 (FT4) levels at 1.61 pg/ml and 0.83 ng/dl, respectively. Luteinizing hormone (LH) and prolactin levels were elevated to 8.65 mIU/ml and 31.74 ng/ml, while testosterone level was reduced to 57.76 ng/dl. Antinuclear antibody (ANA) and antineutrophil cytoplasmic antibody (ANCA) tests were negative. Polymerase chain reaction (PCR) analysis did not detect human immunodeficiency virus (HIV), Epstein–Barr virus (EBV), or human herpesvirus 8 (HHV-8). Serum immunoelectrophoresis identified monoclonal IgG-λ, with an M protein level of 2.53 g/L.

**Table 1:** Laboratory data on admission.

Parameter	Value (reference range)	Parameter	Value (reference range)
Blood cell count		Biochemistry	
WBC, /μl	2,610 (4000–10,000)	AST, IU/L	8 (8–40)
RBC, 10^4^/μl	303 (350–550)	ALT, IU/L	5 (5–40)
Hb, g/L	82 (120–160)	LDH, IU/L	172 (109–245)
MCV, fL	86.5 (82–95)	UA, μmol/L	452 (155–428)
Plt, 10^4^/μl	6.7 (10–30)	BUN, mmol/L	8.5 (1.7–8.3)
		Cre, μmol/L	150 (18–104)
Serological test		β2-MG, μg/ml	6.59 (1.58–2.62)
CRP, mg/L	57 (1–8)		
IL-6, pg/ml	23.23 (<7.0)	Urinalysis	
IgG4, g/L	0.12 (0.07–1.35)	Protein, g/24 h	0.23 (0–0.15)
VEGF, pg/ml	330.69 (0–142)	24 h κ-light-chain, mg/dl	4.19 (0–1.85)
BNP, pg/ml	3,618 (<300)	24 h λ-light-chain, mg/dl	<5.00 (0–5)
M protine, g/L	2.53 (0)		
		OCT	
Endocrine		RNFL thickness (OD), μm	114 (80–100)
PRL, ng/ml	31.74 (2.64–13.13)	RNFL thickness (OS), μm	127 (80–100)
T, ng/dl	57.76 (175–781)		
LH, mIU/ml	8.65 (1.24–8.62)	Coagulation system	
FSH, mIU/ml	8.71 (1.27–19.26)	APTT, s	55.7 (25–43.5)
FT3, pg/ml	1.61 (2.3–4.2)	PT, s	15.8 (11–15)
FT4, ng/dl	0.83 (0.89–1.76)	Fib, g/L	4.31 (2–4)
TT3, ng/ml	0.85 (0.6–1.8)	TT, s	13.4 (14–21)
TT4,μg/dl	4.4 (4.5–10.9)	INR	1.25 (0.8–1.2)
TSH, μIU/ml	5.37 (0.55–4.78)	D-D, μg/ml	2.84 (0.01–0.5)

WBC, white blood cell; RBC, red blood cell; Hb, hemoglobin; MCV, mean corpuscular volume; Plt, platelet; CRP, C-reactive protein; IL-6, interleukin-6; IgG, immunoglobulin G; VEGF, vascular endothelial growth factor; BNP, B-type natriuretic peptide; PRL, prolactin; T, testosterone; LH, Luteinizing hormone; FSH, follicle-stimulating hormone; FT3, free triiodothyronine; FT4, free thyroxin; TT3, total triiodothyronine; TT4, total thyroxin; TSH, thyroid stimulating hormone; AST, aspartate transaminase; ALT, alanine aminotransferase; LDH, lactate dehydrogenase; UA, uric acid; BUN, blood urea nitrogen; Cre, creatinine; β2-MG, β2-microglobulin; OCT, Optical Coherence Tomography; RNFL, retinal nerve fiber layer; OD, oculus dextrus; OS, oculus sinister; APTT, activated partial thromboplastin time; PT, prothrombin time; Fib, fibrinogen; TT, thrombin time; INR, international normalized ratio; D-D, D-dimer.

Imaging studies, including a CT scan, identified long standing cerebral infarcts, bilateral pleural effusion, hydropericardium, ascites, and enlarged breast glandular tissue. PET-CT scan detected multiple small lymphadenopathies in the cervical, axillary, mediastinal, and inguinal regions. Optical Coherence Tomography (OCT) indicated optic papilledema with average retinal nerve fiber layer (RNFL) thicknesses of 114 μm for the right eye and 127 μm for the left. A biopsy of a lymph node from the left cervical region confirmed the presence of Castleman disease (CD).

**Figure 1:**
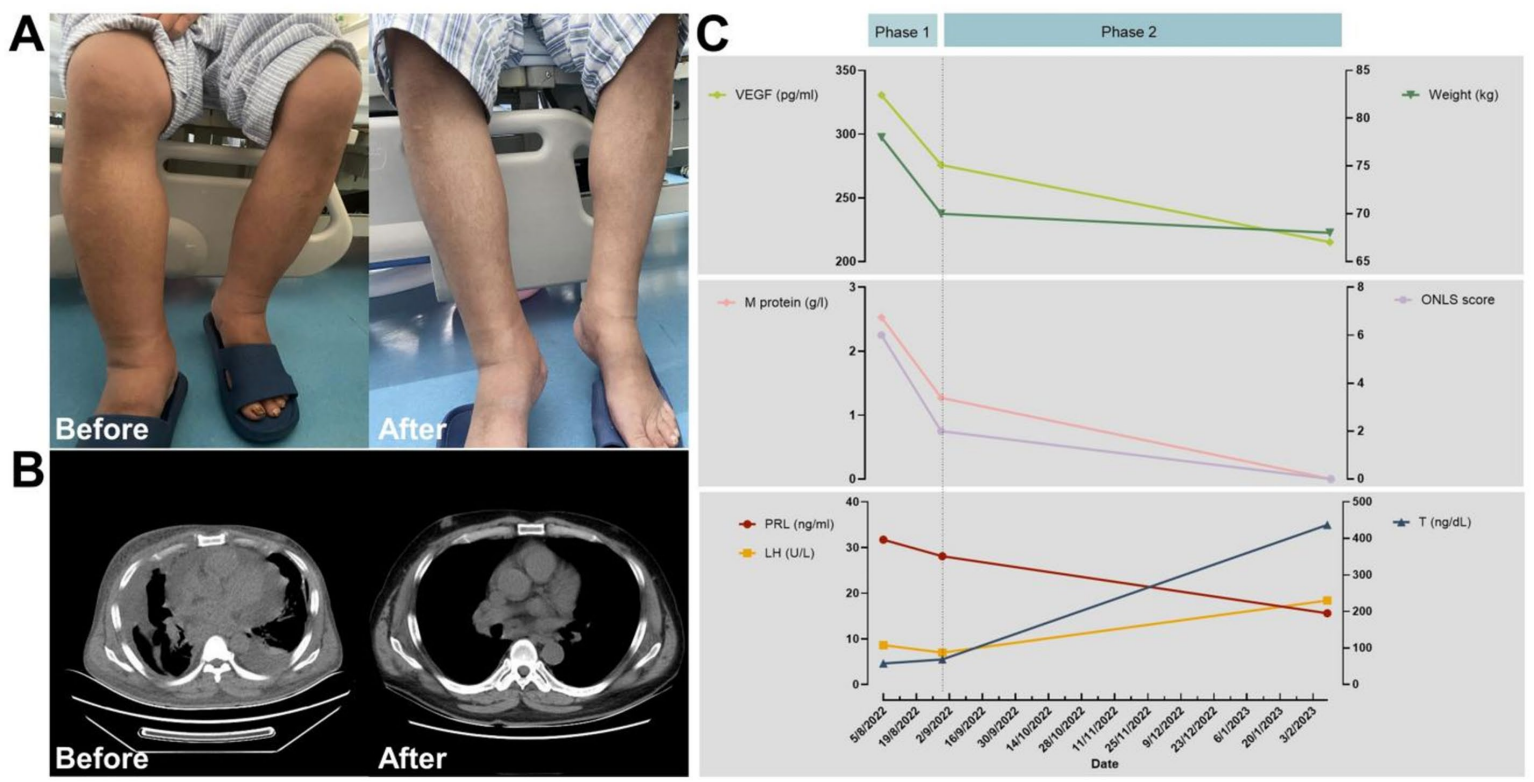
Comparison of patient’s clinical characteristics before and after treatment. **(A)** Effectiveness of treatment for lower limb edema. **(B)** Computed tomography showing fluid accumulation in the serous cavities. **(C)** Treatment course and efficacy; Phase 1: dexamethasone treatment (during the process of diagnosis); Phase 2: three chemotherapy cycles using the Rd. regimen.

The findings of M protein (λ-type), extravascular volume overload, organomegaly (lymphadenopathy), papilledema, endocrinopathy, skin changes, and elevated VEGF levels suggested POEMS syndrome. Meanwhile, thrombocytopenia, elevated serum C-reactive protein, Castleman disease in the lymph nodes, and renal insufficiency pointed towards TAFRO syndrome. For a definitive diagnosis of TAFRO syndrome, POEMS syndrome must be excluded initially, and the diagnosis of Castleman disease necessitates ruling out plasma cell disorders. Although electromyography (EMG) results indicated neurological impairment, the impact of limb edema on these findings could not be overlooked. Clinical evidence leaned more towards a diagnosis of POEMS syndrome. The patient, grappling with severe respiratory and cardiac failure due to excessive extravascular fluid accumulation, initially received dexamethasone intravenously at a dosage of 5 mg/day for 6 days. This treatment led to noticeable improvements in dyspnea and limb edema, as depicted in Figure 1A. Subsequent EMG assessments indicated a significant betterment in peripheral neuropathy symptoms, highlighting the previous EMG’s inconsistency with typical clinical features of peripheral neuropathy and suggesting that severe edema had impacted the initial EMG results. The treatment regimen was then escalated to 10 mg/day of dexamethasone administered intravenously for a total of 13 days, during which the patient’s body weight decreased by 10 kilograms, shown in Figure 1C Phase 1. The patient also received intravenous methylprednisolone (20 mg twice daily) and oral lenalidomide (25 mg/day) (7). This comprehensive treatment approach not only improved the patient’s overall condition but also led to increased blood cell counts, normalized CRP and renal function, and a decrease in serum VEGF levels. The final EMG revealed no significant abnormalities, paving the way for a definitive diagnosis of atypical POEMS syndrome without polyneuropathy (Table 1).

Subsequently, the patient underwent three cycles of chemotherapy following the Rd. regimen (lenalidomide 25 mg/day on days 1–21 and dexamethasone 40 mg/day on days 1, 8, 15, and 22, with each cycle lasting 28 days). Post-chemotherapy, the patient achieved hematologic remission and neuropathic improvement, and though VEGF levels did not fully normalize, sex hormone levels returned to nearly normal. Serosal effusion resolved completely (Figure 1B). The average retinal nerve fiber layer (RNFL) thickness in both eyes returned to normal, decreasing from 127 μm to 108 μm in the left eye and from 114 to 106 μm in the right eye (Supplemental Table 1). The patient then underwent successful autologous peripheral blood stem cell transplantation with a conditioning regimen of melphalan 200 mg/m^2^. Post-transplant, complete remission was achieved in hematologic and serum VEGF levels, with the Overall Neuropathy Limitations Scale (ONLS) score indicating effective neuropathy treatment, and the patient regained normal walking ability.

## 3 Discussion

### 3.1 Differentiation

Initially, the presence of M protein, lymphadenopathy, papilledema, endocrinopathy, and elevated VEGF were indicative of POEMS syndrome. Crucially, polyneuropathy’s presence was a decisive factor in the diagnosis. The patient, however, displayed no clear symptoms of peripheral neuropathy, and examinations of the limb conduction bundles revealed no notable abnormalities. EMG findings post-edema resolution also did not indicate polyneuropathy. Although lymph node biopsies aligned with Castleman’s disease (CD), the M protein’s presence led to the provisional ruling out of CD alone. On the other hand, the symptoms of anasarca, thrombocytopenia, and lymph node CD significantly pointed towards TAFRO syndrome. Nevertheless, this patient lacked typical systemic inflammation markers, such as unexplained fevers above 37.5°C or serum C-reactive protein levels ≥2 mg/dl.

The clinical manifestations of POEMS syndrome are so diverse that the diagnosis can be interfered. A study reported some patients who were diagnosed with POEMS syndrome had an unusual initial presentation of ascites (8). Other studies still reported cases of POEMS syndrome with pleural effusion (9) and optic disc edema (10) as the main symptoms. Following the work of Ryuji Morizane et al. (11), there has been an increase in diagnosing atypical POEMS syndrome cases without polyneuropathy (12) or monoclonal protein (13). Considering the characteristic signs and symptoms, we provisionally diagnosed this patient with atypical POEMS syndrome, lacking polyneuropathy.

### 3.2 Patient in critical condition, and salvage therapy

Upon admission, the patient was critically ill, exhibiting heart failure symptoms, including sedentary respiration. Initial assessments were conducted to rule out cardiac, renal, hepatic, and other organ pathologies. Positive initial EMG results and the presence of M protein suggested the probability of POEMS syndrome. Dexamethasone was administered intravenously, significantly improving the patient’s pleural effusion and extremity edema, aiding the differential diagnosis process.

Chemotherapy using lenalidomide and dexamethasone (Rd regimen) was initiated, leading to significant improvements after three treatment cycles, achieving hematologic and neuropathy remission (14).

### 3.3 Autologous hematopoietic stem cell transplantation

With the goal of achieving comprehensive disease remission and enhancing the patient’s survival outlook (15, 16), the patient underwent ASCT following various chemotherapy courses. This procedure was completed without major complications, resulting in a favorable prognosis.

## 4 Summary

POEMS syndrome is characterized by multisystem impairment, neuropathy, and plasma cell proliferative disorders, whereas TAFRO syndrome is marked by a systemic inflammatory response.

POEMS syndrome presents as a complex condition with key clinical features: peripheral neuropathy’s clinical manifestations and EMG results can be masked by issues such as significant edema and potential sensory deficits following a cerebral infarction in this case. To diagnose, we explored any unusual patterns in electromyography, leading to the rare identification of POEMS syndrome absent of peripheral neuropathy. Glucocorticoids are advised for treating both syndromes, hence their utilization as an initial rescue therapy. This approach has confirmed that, in a case exhibiting symptoms of both POEMS and TAFRO syndromes, the interventions employed can led to a favorable outcome.

## Data Availability

The original contributions presented in the study are included in the article/Supplementary material, further inquiries can be directed to the corresponding authors.

## Supplementary material

SUPPLEMENTARY VIDEO 1 The patient has regained.
