# Supplementary material for: Case report: Atypical POEMS syndrome without polyneuropathy, complicated by borderline TAFRO syndrome
Source: Front Med (Lausanne). 2024 Dec 10;11:1445971. doi: 10.3389/fmed.2024.1445971 (PMC11666388; doi:10.3389/fmed.2024.1445971)
Supplement: Supplementary file 2 [file Table_1.docx]

**Supplementary materials**

|  | **Before treatment** | **After chemotherapy** |
| --- | --- | --- |
| **Serological test** |  |  |
| M protine, g/L | 2.53 | 0 |
| VEGF, pg/mL | 330.69 (0-142) | 215.20 (0-142) |
| BNP, pg/mL | 3618 (<300) | 236 (<300) |
| CRP, mg/L | 57 (1-8) | 3 (1-8) |
| IL-6, pg/mL | 23.23 (<7.0) | 17.3 (<7.0) |
| **Biochemistry** |  |  |
| AST, IU/L | 8 (8-40) | 12 (8-40) |
| ALT, IU/L | 5 (5-40) | 12 (5-40) |
| Cre, μmol/L | 150 (18-104) | 75 (18-104) |
| **Endocrine** |  |  |
| PRL, ng/mL | 31.74 (2.64-13.13) | 13.63 (2.64-13.13) |
| LH, U/L | 8.65 (1.24-8.62) | 18.41 (1.24-8.62) |
| T, ng/dL | 57.76 (175-781) | 437 (175-781) |
| **RNFL thickness** |  |  |
| OD, μm | 114 (80-100) | 106 (80-100) |
| OS, μm | 127 (80-100) | 108 (80-100) |

**Supplemental Table 1. Comparison of key indicators before treatment and after 3 cycles of Rd regimen treatment.** VEGF: vascular endothelial growth factor, BNP: B-type natriuretic peptide, CRP: C-reactive protein, IL-6: interleukin-6, AST: aspartate transaminase, ALT: alanine aminotransferase, Cre: creatinine, PRL: prolactin, LH: Luteinizing hormone, T: testosterone, RNFL: retinal nerve fiber layer, OD: oculus dextrus, OS: oculus sinister
