# Supplementary material for: Case report: Atypical POEMS syndrome without polyneuropathy, complicated by borderline TAFRO syndrome
Source: Front Med (Lausanne). 2024 Dec 10;11:1445971. doi: 10.3389/fmed.2024.1445971 (PMC11666388; doi:10.3389/fmed.2024.1445971)
Supplement: Supplementary file 3 [file Data_Sheet_1.PDF]

CARE case report guidelines

CARE Checklist of information to include when writing a case report

(cc) BY-NC-ND

Topic	Item	Checklist item description	Reported on Line
Title	1	The diagnosis or intervention of primary focus followed by the words "case report"	Title,page1
Key Words	2	2 to 5 key words that identify diagnoses or interventions in this case report, including "case report"	Key words,page3
Abstract (no references)	3a	Introduction: What is unique about this case and what does it add to the scientific literature?	Introduction, page4
	3b	Main symptoms and/or important clinical findings	Paragraph1-4,page5
	3c	The main diagnoses, therapeutic interventions, and outcomes	Paragraph1-2,page6
	3d	Conclusion—What is the main "take-away" lesson(s) from this case?	Discussion,page7
Introduction	4	One or two paragraphs summarizing why this case is unique (**may include references**)	Summary,page8
Patient Information	5a	De-identified patient specific information.	Paragraph1,page5
	5b	Primary concerns and symptoms of the patient.	Paragraph1,page5
	5c	Medical, family, and psycho-social history including relevant genetic information	Paragraph2,page5
	5d	Relevant past interventions withoutcomes	Paragraph2,page5
Clinical Findings	6	Describe significant physical examination (PE) and important clinical findings.	Paragraph2,page5
Timeline	7	Historical and current information from this episode of care organized as a timeline	Paragraph1,page5
Diagnostic Assessment	8a	Diagnostic testing (such as PE, laboratory testing, imaging, surveys).	Paragraph2-4,page5
	8b	Diagnostic challenges (such as access to testing, financial, or cultural)	Paragraph1,page6
	8c	Diagnosis (including other diagnoses considered)	Paragraph1,page6
	8d	Prognosis (such as staging in oncology) where applicable	Paragraph2,page6
Therapeutic Intervention	9a	Types of therapeutic intervention (such as pharmacologic, surgical, preventive, self-care)	Paragraph1-2,page6
	9b	Administration of therapeutic intervention (such as dosage, strength, duration)	Paragraph1-2,page6
	9c	Changes in therapeutic intervention (with rationale)	Figure1
Follow-up and Outcomes	10a	Clinician and patient-assessed outcomes (if available)	Figure1
	10b	Important follow-up diagnostic and other test results	Paragraph1-2,page6
	10c	Intervention adherence and tolerability (How was this assessed?)	Paragraph2,page6
	10d	Adverse and unanticipated events	Paragraph1,page6
Discussion	11a	A scientific discussion of the strengths AND limitations associated with this case report	Differentiation,page7
	11b	Discussion of the relevant medical literature **with references**.	References,page10
	11c	The scientific rationale for any conclusions (including assessment of possible causes)	Summary,page8
	11d	The primary "take-away" lessons of this case report (without references) in a one paragraph conclusion	Summary,page8
Patient Perspective	12	The patient should share their perspective in one to two paragraphs on the treatment(s) they received	Paragraph2,page6
Informed Consent	13	Did the patient give informed consent? Please provide if requested	Yes √☐ No ☐
